# Continuous adsorption of natural organic matters in a column packed with carbon nanotubes

**DOI:** 10.1186/2052-336X-11-14

**Published:** 2013-07-03

**Authors:** Ali Naghizadeh, Simin Nasseri, Amir Hossein Mahvi, Ramin Nabizadeh, Roshanak Rezaei Kalantary, Alimorad Rashidi

**Affiliations:** 1 Department of Environmental Health Engineering, School of Public Health, Tehran University of Medical Sciences, Tehran, Iran; 2 Center for Water Quality Research (CWQR), Institute for Environmental Research (IER), Tehran University of Medical Sciences, Tehran, Iran; 3 Center for Air Quality Research (AWQR), Institute for Environmental Research (IER), Tehran University of Medical Sciences, Tehran, Iran; 4 Nanotechnology Research Center, Research Institute of Petroleum Industry (RIPI), Tehran, Iran

**Keywords:** Carbon nanotubes, Natural organic matters, Adsorption capacity

## Abstract

In the present study, continuous adsorption experiments were carried out in an adsorption column to survey the efficiency of the carbon nanotubes (CNTs) for removal of natural organic matters (NOMs) from aqueous solution. Parameters such as mass of CNTs, initial NOMs concentration were evaluated and also the breakthrough curves were obtained. Experiments performed with various initial NOMs concentration and various CNTs masses. The breakthrough period was longer at lower initial NOMs concentration. Increase of the initial NOMs concentration, expectedly, resulted in the faster saturation of the CNTs bed. The adsorption capacities for multi wall carbon nanotubes (MWCNT) and single wall carbon nanotubes (SWCNT) in highest initial NOMs concentration were 53.46 and 66.24 mg/g, respectively. The effect of amount of CNTs on breakthrough time and volume of treated water was investigated and resulted that with an increase in the mass of CNTs, breakthrough time occurs very late and the volume of treated water increased. These findings suggested that CNTs present a great potential in removal of NOMs from aqueous solutions.

## Background

Natural organic matters (NOMs) is present in all drinking water sources and is a complex mixture of compounds formed from the breakdown of plant and animal material in the environment [1]. NOMs cause problems in the production of drinking water. It has an adverse effect on the aesthetic water quality and may result in biofouling of pipelines with negative hygienic consequence [2]. It has also been demonstrated that the NOM is the basis for the production of the potentially hazardous disinfection by-products (DBPs) [3]. Thus, is has to be removed from drinking water more efficiently.

NOMs can be removed from water by a number of different treatment processes, the most common processes are coagulation and flocculation, ion exchange, nanofiltration, reverse osmosis and adsorption [4]. The major disadvantage of Coagulation and flocculation processes is that NOMs with low molar mass (LMM) and intermediate molar mass (IMM) cannot be removed efficiently when this method is used [5]. On the other hand, slow kinetics is the major restriction in the use of ion exchange process for removal of NOMs. The costs of membrane filtration processes such as nanofiltration and reverse osmosis, however, have been relatively high and its use, therefore, is restricted to special cases [6].

Adsorption process using activated carbon as adsorbents has been widely used for removal of NOMs from water [7-9].

The discovery of carbon nanotubes (CNTs) by Iijima [10] added a new member to carbon family. Their novel and unique chemical and physical properties attract many researchers and show a great potential for wide applications such as nanotechnology, electronics, optics, water treatment, and other fields of materials science. The relative large specific surface area (SSA), another important property of CNTs, enables them to become candidate for adsorption of gas [11], metal ions [12] and organic compounds [13].

In the previous study, CNTs have shown exceptional adsorption capability and high adsorption efficiency for various organic contaminants such as dioxin [14], benzene [15], 1,2-dichlorobenzene [16], trihalomethanes [17] and polycyclic aromatic hydrocarbons (PAHs) [18].

All these studies mentioned above show that CNTs are promising adsorbents for organic pollutants. The driving forces for interactions between NOMs with activated carbon (AC) and CNTs could be similar since both AC and CNTs are composed of graphene sheets thus similar chemical composition. However, due to distinct arrangement of graphene sheets in AC and CNTs thereby dissimilar surface and structural characteristics, the interaction mechanisms between NOMs and these two types of carbonaceous materials could be different. However, because removal of NOMs by CNTs has rarely been discussed, we carried out this study.

In the present study, multiwall carbon nanotubes (MWCNT) and single wall carbon nanotubes (SWCNTs) were used for NOMs removal. Continuous adsorption experiments were conducted to understand and quantify the effect of influencing parameters such as mass of adsorbent, initial NOMs concentration on breakthrough curve.

## Methods

### CNTs preparation

CNTs were prepared by Chemical vapor deposition (CVD) method in Research Institute of Petroleum Industry (RIPI), Tehran, Iran. In order to purification and removing the metal nanocatalysts from carbon nanotubes, the final products were dissolving in 37% hydrochloride acid solution for about 16 h at ambient temperature and then washed several times with distilled water until the pH of the solution reached approximately neutral. Treated CNTs were dried in vacuum at 40°C overnight. For eliminate the amorphous carbons, all of the purified CNTs were placed in the furnace at 400°C for 30 min.

### Continuous experiments

A stock solution of 1000 mg/L of NOMs was prepared by dissolving special amounts of humic acid in water. This solution was diluted as required to obtain standard solutions containing 3, 5 and 10 mg/L of NOMs.

**Figure 1:**
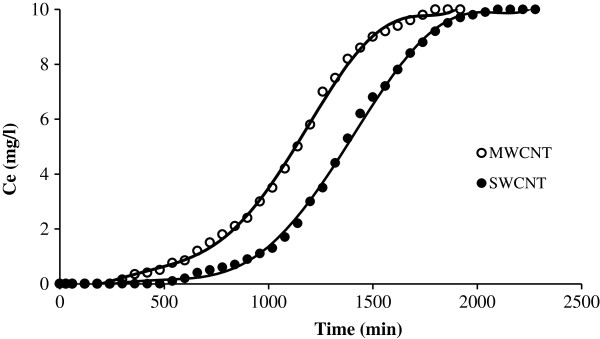
**Continuous adsorption of NOMs on CNTs at initial concentration of 10 mg**/**L.**

Continuous fixed-bed experiments were performed to remove NOMs from aqueous solutions using CNTs as adsorbent. The schematic diagram of the experimental setup is shown in Figure 1. The fixed-bed column was made of stainless steel.

First, 500 mL of distilled water was passed through the packed bed to make the packing more compact packing. Stock solutions of NOMs were allowed to flow in down flow mode through the fixed-bed of CNTs controlled by a valve. The parameters varied in the continuous experiments were mass of adsorbent (bed-height), and inlet NOMs concentration. The flow rate was maintained constant using a liquid rotameter (5mL/min). The fixed mass of the adsorbent and the stock solution of initial NOMs concentration were used to maintain the higher accuracy in column experiments. The continuous experiments were carried out at an optimum pH value of 3 obtained by batch experiments.

In the present work, TOC analyzer (TOC-VCSH, Shimadzu, Japan) was used for the analysis of NOMs in the aqueous solutions. The total quantity of adsorbed NOMs (q_t_, mg) in the column for a given inlet NOMs concentration and flow rate was calculated from Equation 1:

(1) $$q_{t}=\frac{{\mathrm{QA}}_{C}}{1000}=\frac{Q}{1000}\int_{t=0}^{{t=t}_{\text{total}}}C_{\mathrm{ad}}\mathrm{dt}$$

Where q_t_ is the amount of NOMs adsorbed by CNTs (mg/g), A_c_ is the area under the breakthrough curve (that was obtained by plotting the adsorbed concentration versus time (t)); C_ad_ is the NOMs adsorbed concentration (mg/L); Q is the flow rate of NOMs solution (mL/min).

The empty bed residence time (EBRT) is the time required for the liquid to fill the empty column. The EBRT is given by Equation 2:

(2) $$\text{EBRT}=\frac{\text{Bed}\;\text{Volume}}{\text{Volumetric}\;\text{flow}\;\text{rate}\;\mathrm{of}\;\text{the}\;\text{liquid}}$$

## Results

**Figure 2:**
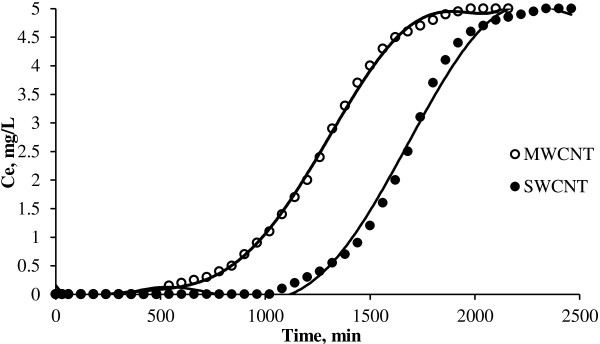
**Continuous adsorption of NOMs on CNTs at initial concentration of 5 mg**/**L.**

**Figure 3:**
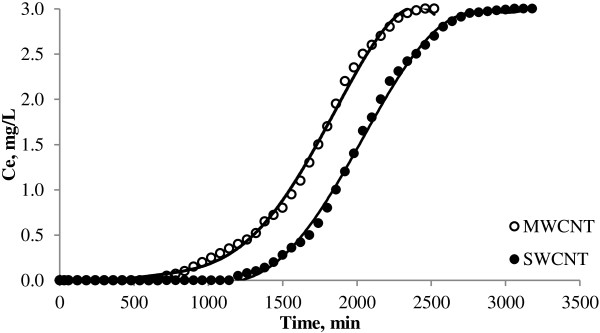
**Continuous adsorption of NOMs on CNTs at initial concentration of 3 mg**/**L.**

**Figure 4:**
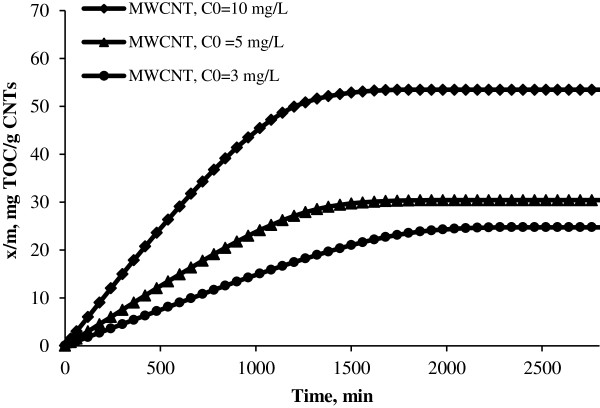
Adsorption capacities of MWCNT in different initial concentrations.

**Figure 5:**
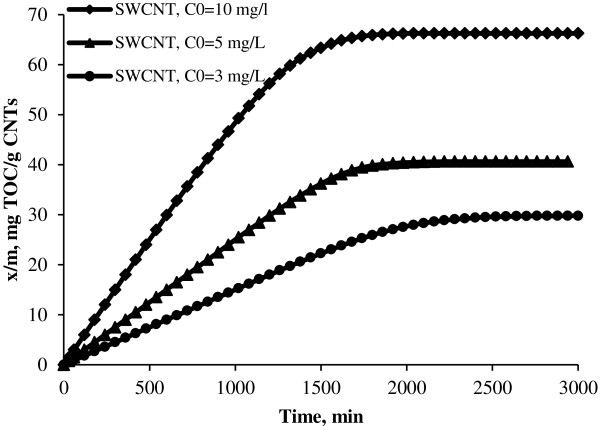
Adsorption capacities of SWCNT in different initial concentrations.

**Figure 6:**
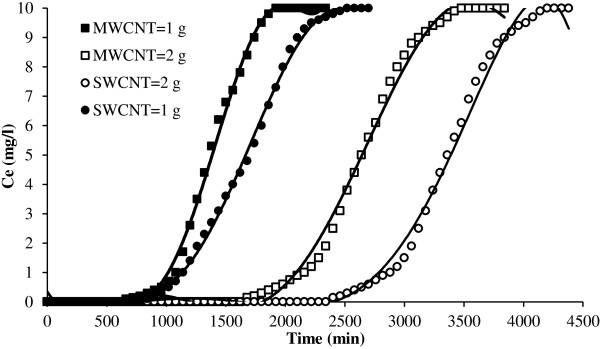
Effect of masses of CNTs on breakthrough curve for NOMs removal.

The breakthrough curves for MWCNT and SWCNT obtained at 10 mg/L initial NOMs concentrations are shown in Figure 1. Also the curves of continuous adsorption of NOMs on CNTs in 5 and 3 mg/L initial concentration has been showed in Figures 2 and 3 respectively. Different parameters for the NOMs removal using CNTs in a fixed-bed adsorption column for different operating conditions are shown in Table 1. Figure 4 shows the adsorption capacities of multi wall carbon nanotubes in different initial concentrations of NOMs. The adsorption capacities of multiwall carbon nanotubes also could be seen in Figure 5. Figure 6 shows the performance of breakthrough curves at different values of mass of adsorbent.

**Table 1:** **Different parameters for the NOMs removal using CNTs in a fixed**-**bed adsorption column for different operating conditions**

**CNTs types**	**Initial NOMs concentration (mg/L)**	**Break point time parameters**			**Saturation point time parameters**		
		**V**_**bp**_**, mL**	**t**_**bp**_**, min**	**q**_**bp**_**, mg/g**	**V**_**sat**_**, mL**	**t**_**sat**_**, h**	**q**_**sat**_**, mg/g**
MWCNT	10	2400	480	23.58	9000	1800	53.46
	5	3300	660	16.32	9900	1980	30.40
	3	4500	900	13.39	12300	2460	24.75
SWCNT	10	3600	720	35.64	10500	2100	66.24
	5	6000	1200	29.82	11700	2340	40.63
	3	10200	1380	27.96	15300	3060	29.77

V_**bp **_and V_**sat**_: volumes of treated water up to breakthrough point time and saturation point time respectively.

t_**bp **_and t_**sat**_: Breakpoint time and saturation point time respectively.

q_**bp **_and q_**sat**_: adsorption capacities in breakpoint time and saturation point time respectively.

## Discussion

In this study, fixed-bed adsorption studies were used to evaluate the behavior of NOMs removal onto the single and multi wall carbon nanotubes.

### Effect of initial noms concentration

According to Figures 1, 2 and 3, as the initial NOMs concentration was increased from 3 to 10 mg/L, the break point time for MWCNT decreased from 900 to 480 min. Also for the SWCNT, as the initial concentration of NOMs was increased from 3 to 10 mg/L, the break point time decreased from 1380 to 720 min. Similar finding have been reported by Gupta and Babu who evaluated the removal of chromium by a fixed bed adsorption column and pointed out that with increasing the initial concentration, the break point time decreased [19].

The most important criterion in the design of fixed-bed adsorption systems include the estimation of the shape of the breakthrough curve and the appearance of the breakpoint time which determine the operation life span of the bed. Previous study pointed that the breakpoint time is the time of adsorption when the effluent concentration from the column was about 1-5% of the influent concentration [20].

Regarding to the Table 1 and also according to the total capacity of the CNTs in the column, the total time for adsorption, was found to be decreased from 2460 to 1800 min with an increase in the initial NOMs concentration from 3 to 10 mg/L. Therefore, a change in the initial NOMs concentration has a significant influence on the characteristics of the breakthrough curve. As shown in Table 1 and Figures 1, 2 and 3, with increasing the initial NOMs concentration from 3 to 10 mg/L, the volume of treated water decreased from 4500 to 2400 mL for MWCNT and from 10200 to 3600 for SWCNT.

The increase in the initial NOMs concentration led to reach bed saturation earlier and the break point time was quickly obtained due to the relatively slower transport because of a decrease in diffusion coefficient and the decreased mass transfer coefficient at low NOMs concentration [21]. At higher initial concentration of natural organic matters, adsorption sites quickly saturated and result in decrease in the breakthrough time. It is observed that the adsorbent gets saturated faster at higher concentrations of adsorbate due to the higher rate of adsorbent exhaustion at higher NOMs concentration. On the other hand, for a low initial NOMs concentration, breakthrough occurs very late and the surface of the adsorbents is saturated with NOMs at a relatively longer time. This fact is probably associated with the availability of adsorption sites around or inside the adsorbent particles that are able to capture the NOMs at a lesser retention time. Other studies have also reached to the same results [22].

As shown in Figures 4 and 5, the longer time needed to reach equilibrium for lower initial NOMs concentration may be explained by the fact that diffusion mechanisms control the adsorption of NOMs onto CNTs. Reid et al. indicated that the mass diffusivity decreases with decreasing concentration under very dilute solution and causes the decrease in diffusion flux of adsorbate onto the surface of the adsorbent [23]. This can be interpreted that if we choose constant mass of adsorbent and constant volume of solution and low concentration of natural organic matters in the solution, therefore, the time needed to adsorbate reached to adsorption sites is higher than when we used high NOMs concentrations.

### Effect of carbon nanotubes mass

The amount of CNTs used in the removal of NOMs from aqueous solution is critical for the application of these nonomaterials in NOMs elimination. If the masses of CNTs used in the removal of NOMs are high, then the cost of using CNTs as adsorbent is high and they cannot be used extensively. According to Figure 6 with an increase in the masses of CNTs, the capacities of single and multi wall carbon nanotubes to adsorb NOMs increases which results in a delay to obtain the breakthrough time and also increase in volume of treated water. This may be due to the increase in the adsorbent surface area with increase in the adsorbent amount which provides more binding sites for the adsorption. Other studies have also reached to the same results [24].

### Mechanism of noms adsorption

Different mechanisms may act simultaneously on natural organic chemical and CNT interactions, such as hydrophobic interactions, π-π bonds, electrostatic interactions, and hydrogen bonds. Yang and Xing concluded that the maximum adsorption capacity of a given organic pollutant on CNTs depended on the CNT surface area; surface functional groups on CNTs, the pores in CNT aggregates and surface curvature and defects of CNT monomers [25]. The NOM might provide sterically and electrostatically stable surfaces after adsorption to CNTs. NOMs such as humic acids maybe alter their surface physicochemical properties and enhance their stabilization in water by increasing the steric repulsion and reduce the van der Waals forces between particles.

## Conclusion

In this study feasibility of CNTs column adsorption in removal of NOMs was investigated. The parameters such as initial NOMs concentration and CNTs dosage were studied. The results showed that with an increase in the mass of CNTs and initial concentrations of NOMs, the breakthrough time occur very late and the volume of treated water increases. The adsorption capacities of SWCNTs and MWCNTs in removal of NOMs were significant.

## Competing interests

The authors declare that no conflict of interest.

## Authors’ contributions

All authors contributed to the manuscript. All persons listed as authors have read, contributed to preparing the manuscript and attest to the validity and legitimacy of the data and its interpretation, and agree to its submission to “Iranian Journal of Environmental Health Science & Engineering”. No person more than the authors listed have contributed significantly to its preparation. All authors read and approved the final manuscript.
